# Overexpression of Mitochondrial Uncoupling Protein 2 Inhibits Inflammatory Cytokines and Activates Cell Survival Factors after Cerebral Ischemia

**DOI:** 10.1371/journal.pone.0031739

**Published:** 2012-02-14

**Authors:** Bryan Haines, P. Andy Li

**Affiliations:** 1 The Buck Institute for Research on Aging, Novato, California, United States of America; 2 Department of Pharmaceutical Sciences, Biomanufacturing Research Institute and Technological Enterprise (BRITE), North Carolina Central University, Durham, North Carolina, United States of America; Julius-Maximilians-Universität Würzburg, Germany

## Abstract

Mitochondria play a critical role in cell survival and death after cerebral ischemia. Uncoupling proteins (UCPs) are inner mitochondrial membrane proteins that disperse the mitochondrial proton gradient by translocating H^+^ across the inner membrane in order to stabilize the inner mitochondrial membrane potential (ΔΨ_m_) and reduce the formation of reactive oxygen species. Previous studies have demonstrated that mice transgenically overexpressing UCP2 (UCP2 Tg) in the brain are protected from cerebral ischemia, traumatic brain injury and epileptic challenges. This study seeks to clarify the mechanisms responsible for neuroprotection after transient focal ischemia. Our hypothesis is that UCP2 is neuroprotective by suppressing innate inflammation and regulating cell cycle mediators. PCR gene arrays and protein arrays were used to determine mechanisms of damage and protection after transient focal ischemia. Our results showed that ischemia increased the expression of inflammatory genes and suppressed the expression of anti-apoptotic and cell cycle genes. Overexpression of UCP2 blunted the ischemia-induced increase in IL-6 and decrease in Bcl2. Further, UCP2 increased the expression of cell cycle genes and protein levels of phospho-AKT, PKC and MEK after ischemia. It is concluded that the neuroprotective effects of UCP2 against ischemic brain injury are associated with inhibition of pro-inflammatory cytokines and activation of cell survival factors.

## Introduction

Hyperpolarization of the mitochondrial membrane potential precedes reactive oxygen species production and cell death in cultured neurons and astrocytes exposed to oxygen and/or glucose deprivation [1], [2]. Inhibiting hyperpolarization protected neuronal cells from oxidative stress-induced cell death [3]. It is likely that mitochondrial hyperpolarization places pressure on the electron transport chain increasing the chance for incomplete oxygen reduction when there is a persistent flow of electrons from NADH and FADH2. This condition occurs after recirculation or re-oxygenation following stroke in vivo or hypoxia in vitro.

Uncoupling proteins (UCPs) are located in the inner mitochondrial membrane and function to transport protons into the mitochondrial matrix. UCPs were first described for the ability to generate heat without shivering in brown adipose tissue (BAT) [4]. Subsequent studies revealed that reduction of the proton motor force across the mitochondrial inner membrane by UCP2 decreased the formation of reactive oxygen species [5]. A small reduction in the mitochondrial membrane potential induced by mild uncoupling has a significant effect in attenuating reactive oxygen species (ROS) production [6]. UCP2 is ubiquitously expressed in all tissues with more levels in the brain and skeletal muscle at levels up to 1000 times less than UCP1 in BAT [7], [8]. Proposed functions of UCP2 include preventing the formation of ROS and atherosclerosis, participation in inflammation, body weight regulation, adaptive thermogenesis and aging [9].

The majority of studies have demonstrated increasing UCP2 is neuroprotective; however, it is still a matter of debate. Up-regulation of UCP2 has been reported to reduce neuronal damage in cerebral stroke, traumatic brain injury, epilepsy and Parkinson's models [10]–[13]. Neuroprotective ischemic pre-conditioning and ischemic post-conditioning up-regulate UCP2 expression [14]. Neuroprotection by ischemic post-conditioning is partially dependent on AKT phosphorylation and involves the mitogen-activated protein kinase (MAPK) and protein kinase-C (PKC) pathways [15]. Neuroprotection by ischemic pre-conditioning and hypothermia is dependent on phosphorylated AKT [16], [17]. The role of ERK1/2 in the MAPK pathway during cerebral ischemia is in debate; however, ERK1/2 is transiently increased in response to neuroprotective estrogen, hypothermia, ischemic pre-conditioning and post-conditioning [18], [19]. Ischemic pre-conditioning and post-conditioning prevent the decrease in phosphorylated εPKC after stroke [15].

Oxidative stress and delayed inflammation are critical factors in facilitating neuronal death after cerebral ischemia-reperfusion injury [20]. It has been established that ROS production is increased after cerebral ischemia and reperfusion and such increases initiate expression of inflammatory cytokines [21]. These, in turn, stimulate innate inflammation to generate more ROS, creating a positive feedback mechanism. Tumor necrosis factor-α (TNF-α) and interleukin-6 (IL-6) are up-regulated after ischemic injury [22]. TNF-α has a divergent role in brain injury. Blocking TNF-α by antibodies, TNF binding protein or genetic knockout protects against cerebral ischemia [22], [23]. In contrast to those findings, administering TNF-α before cerebral ischemia is neuroprotective, and ischemic pre-conditioning increases levels of TNF-α to activate the neuroprotective functions behind Nf-κB [22], [23].

The goals of this study were to explore the mechanisms behind UCP2-mediated neuroprotection after transient focal ischemia. We evaluated changes in gene and protein expression related to inflammation and p53 associated proteins. One hour of middle cerebral arterial occlusion with 24 hours of reperfusion was utilized to determine infarct volumes, changes in gene expression and changes in protein expression. Our results confirmed previous studies that demonstrated up-regulating UCP2 reduces infarction after transient focal ischemia [12]. Overexpressing UCP2 reduces the levels of IL-6 mRNA, but not Tnf-α after ischemia. UCP2 Tg mice had increased protein levels of phospho-Ser AKT 473, Heat Shock Protein 90, Protein Kinase C, MAP Kinase Kinase, and MAP Kinase Kinase 4 compared with wild-type controls after ischemia.

## Materials and Methods

### Animals

Fifty-seven mice (30 wild-type and 27 UCP2 Tg) were randomly assigned into control and ischemic group for cerebral vasculature examination, infarct volume measurement, PCR array and protein array studies. Number of animals used in each experiment is given in figure legend. All procedures were performed in strict compliance with the National Institutes of Health guidelines for animal research and were approved by the Institutional Animal Care and Use Committee. UCP2/3 transgenic overexpressing mice were generated by overexpressing human UCP2 and UCP3 within the native promoter [24]. Uncoupling proteins 2, 4 and 5 are the predominant endogenous isoforms found in brain the brain [8]. Because the current experiments were performed in brain tissue and UCP3 is not expressed in brain,, this mouse genotype will be referred to as UCP2 Tg. Mice were backcrossed onto a C57/Bl6 background. Mice were fasted overnight with free access to water. Anesthesia was induced with 3% and maintained at 1–1.5% isoflurane in 30% oxygen and 70% nitrous oxide. Body and head temperatures were maintained between 36.5–37.5°C by a combination of a heating blanket and a lamp. Animals with a fasting blood glucose level between 4–6 mM were used for the experiment. Pre-ischemic blood pressure was 100–120 mmHg with no statistical difference between UCP2 Tg and wild-type animals.

### Anatomy of the Middle Cerebral Artery and Circle of Willis

Naïve wild-type and UCP2 Tg mice (n = 3 in each group) were deeply anesthetized and transcardially perfused with 2% India ink in 20% gelatin in saline. Mice were decapitated after 30 seconds and brains were removed and fixed with 4% paraformaldehyde. Brain images were captured using a Leica dissecting scope (Leica Microsystems, Wetzlar, Germany).

### Ischemic model

Transient middle cerebral artery occlusion (MCAO) was induced in 9 wild-type and 7 UCP2 Tg mice as described before [25]. Briefly, a nylon monofilament (Doccol, Redlands, CA, USA) coated with silicon, size 6-0, was inserted into the common carotid artery to the internal carotid artery in order to block the middle cerebral artery. Mice were revived and behavior was observed for neurological defect to confirm the successfulness of MCAO. The degree of functional deficit at 30 min post occlusion was scored using a 5-point Bederson's scale [26]. Briefly, scale 0, no deficit; 1, mild forelimb weakness; 2, severe forelimb weakness and consistently turns to side of deficit when lifted by tail;3, compulsory circling; 4, unconscious; and 5, dead. Three wild-type and 2 UCP2 Tg mice with a Bederson's score less than 2 were excluded from the study. After 1 hour of ischemia, mice were re-anesthetized and the filament was removed to restore blood flow.

### Measuring Brain Infarction

Infarct volume was determined by using 2% 2, 3, 5-Triphenyltetrazolium chloride (TTC) staining. After 1 hour of MCAO and 24 hours of reperfusion, mice were deeply anesthetized with 5% isoflurane and transcardially perfused with ice cold saline. The brains were removed and sectioned coronally at 1 mm thick using a brain matrix (Harvard Apparatus, Holliston, MA, USA), and incubated in 2% TTC for 15 minutes at room temperature. Brain slices were then fixed in 4% paraformaldehyde, scanned (Hewlett Packard, Palo Alto, CA, USA) into a computer, and quantified using NIH imaging software (rsb.info.nih.gov/nih-image).

### PCR Array

At 24 hours reperfusion, both control and ischemic animals from wild-type and UCP2 Tg (n = 4 in each group) were decapitated after being deeply anesthetized with 5% isoflurane. The brains were extracted within 30 seconds, frozen in liquid nitrogen and stored at −80°C for later dissection. A peripheral area of the ipsilateral cortex (equivalent to the ischemic penumbra area in this model) was dissected in a −20°C glove box. RNA was isolated by Mini RNeasy Columns (Qiagen, Rockville, MD, USA), treated with DNase 1for 30 minutes at 37°C and, then, deactivated with 25 mM EDTA and incubation at 65°C and stored at −80°C. cDNA was synthesized using 1 µg of RNA Superscript III and oligo dT (Invitrogen, Carlsbad, CA, USA). RNA and primers were annealed at 65°C for 5 min. The Superscript enzyme was added and incubated for 50 min at 50°C. The reaction was terminated by incubating at 85°C for 5 min and stored at −20°C. Ninety-six-well PCR plates pre-spotted with oligonucleotides for p53 associated genes (SuperArray, Frederick, MD, USA) were used with SYBR Green RT-PCR master mix (Invitrogen) and run on the ABI 9600 (Applied Biosystems, Foster City, CA, USA). One cDNA prep was used for each array plate. The sample was denatured and polymerase enzyme was heat activated for 10 min at 95°C, 40 cycles of 15 sec 95°C and 1 min at 60°C amplified the transcript and data was collected at each cycle. Crossing threshold (Ct) values were manually set at 2.0 and were normalized to the housekeeping genes β-actin and HPRT1. The PCR array included control wells with no template and wells with no primers.

### p53 Protein Array

A separate set of animals was used for the antibody protein array study. At 24 hours reperfusion, mice were decapitated under deep anesthesia with 5% isoflurane. The brains were excised (n = 4 in each group), frozen in liquid nitrogen and stored at −80°C for later dissection. A peripheral area of the ipsilateral cortex was dissected in a −20°C glove box. The brains were homogenized using a tissue homogenizer (Cole-Palmer, Vernon Hills, IL, USA) at 14,000 rpm in the Sigma Protein Isolation Buffer (Sigma, St. Louis, MO, USA) containing 1 m M EDTA (Sigma), 5 mM DTT (Sigma) and protease inhibitors (Thermo Scientific, Rockford, IL, USA). The homogenates were centrifuged at 750 g for 15 min to separate the nuclear fraction from the cytosolic and mitochondrial fractions. The supernatant containing the cytosolic and mitochondrial proteins was used for cytokine quantification. Protein was quantified using the A280 protein quantification program on the NanoDrop 2000 (Thermo Scientific). 1 mg of total protein (at 1 mg/mL) was directly labeled with Cy5 dye reagent (Sigma) in 100 mM carbonate-bicarbonate buffer pH 9.6, excess dye was removed by a SigmaSpin column, incubated on the protein array slide (Sigma), and signal was detected using a fluorescent array reader (Agilent, Santa Clara, CA, USA). Protein levels were normalized to cytoketerin on the array.

### Data analysis

Experiments were carried out by BH and data were analyzed by PAL without knowing the experimental conditions. T-test was used to analyze infarct volume between the two species of animals. PCR data were analyzed using analysis RT^2^ Profiler™ PCR Array Data Analysis Excel Template provided by SuperArray and protein array data were analyzed using Data Analysis Workbook for the Panorama p53 Array provided by Sigma. P value<0.05 was considered statistically significant.

## Results

### Cerebral Vasculature

**Figure 1:**
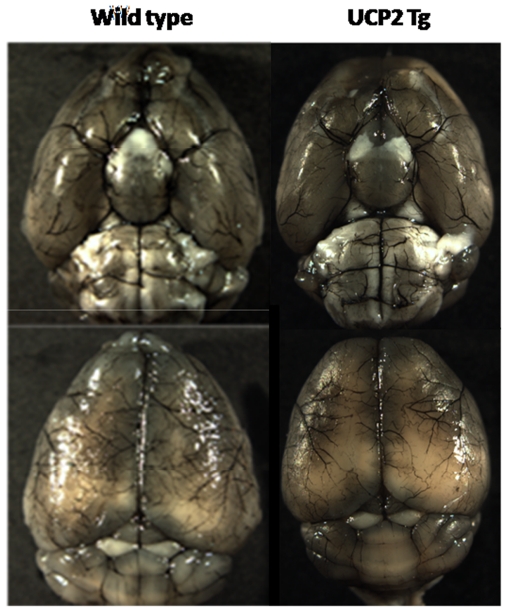
Major cerebral vasculature detected by perfusion of carbon black in wild-type and UCP2 Tg mice. The Circle of Willis, anterior cerebral arteries, middle cerebral arteries and posterior arteries all appear normal in both wild-type and UCP2 Tg animals (n = 3 for each group).

To evaluate if transgenically overexpressing UCP2 in the brain caused a phenotypic change in the cerebral vasculature, we transcardially injected carbon black ink and imaged the cerebral blood vessels (**Fig. 1**). Both wild-type and UCP2 Tg mice demonstrated intact and correct alignment of the Circle of Willis, anterior cerebral arteries, middle cerebral arteries and posterior arteries with no remarkable difference.

### Infarct volume

**Figure 2:**
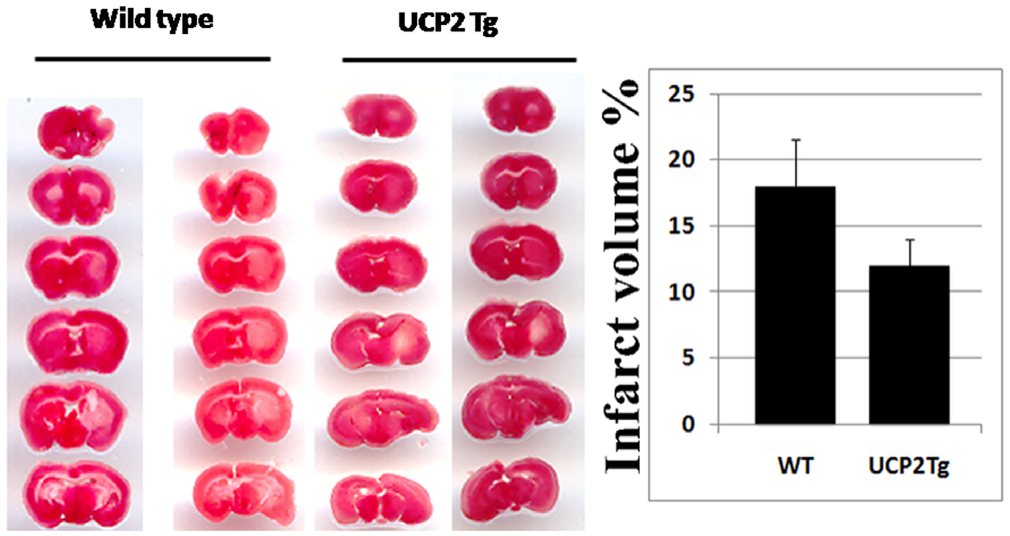
Infarct volume at 24 hours of recovery after 1 hour MCAO. **A,** Representative TTC stained brain sections depict the infarct area (white color). **B**, percentage of infarct volume per hemisphere. Data were collected from 6 wild-type and 5 UCP2 Tg mice. * P<0.05, Student *t* test.

One hour middle cerebral arterial occlusion with 24 hours reperfusion induced a mild brain damage located predominantly in the caudate-putamen in wild-type mice. Infarct volume was measured as a percentage per hemisphere. Mice overexpressing UCP2 in the brain had significantly less damage after transient focal ischemia (**Fig. 2**). Thus the infarct volume was reduced from 18% in wild-type to 12% in UCP2 Tg mice (33% reduction, p<0.01).

### Alteration of Gene Expression

To determine the mRNA expression profile in the ischemic penumbra area of wild-type and UCP2 Tg mice after transient focal ischemia, the p53 PCR array (Qiagen, Frederick, MD) was used to measure transcript levels of 84 genes using quantitative PCR (ABI 7300). Housekeeping genes Hprt1 and β-actin were used to normalize results. The original comparisons were made by comparing wild-type MCAO over wild-type sham, UCP2 Tg MCAO over UCP2 Tg sham, and UCP2 Tg sham over wild-type sham. Because of no significant change in gene expression between UCP2Tg sham and wild-type sham and variation in the UCP2Tg sham, we decided to use wild-type sham as controls. Data are summarized in Table 1. After transient focal ischemia in wild-type mice compared with wild-type sham controls,, cytokines IL-6 (23.36 fold, p = 0.05) and Tnf-α (20.19 fold, p<0.001) are increased significantly. Cell cycle genes: Chek1 (1.78, p = 0.024), Esr1 (3.35 fold, p = 0.022), Myc (3.93 fold, p = 0.039), and RelA (1.77 fold, p = 0.046), were also increased less profoundly. The anti-apoptotic gene Bcl-2 (−1.98 fold p = 0.021) was decreased in the wild-type ischemic penumbra compared with sham controls. Cell cycle genes Cdk4 (−5.00 fold, p = 0.18), Cnng2 (−1.76 fold, p = 0.014) and Ccnh (−1.42, p = 0.074) were also decreased in the wild-type ischemic penumbra. In UCP2 Tg mice, the cytokines IL-6 (7.34 fold, p = 0.037) and Tnf-α (24.04 fold, p = 0.001) were increased after MCAO compared to wildtype sham controls, but the increase in IL-6 was much less profound than in wild-type animals (7.3-fold versus 23.4-fold). Chek1 (2.24 fold, p = 0.008), Esr1 (1.39 fold, p = 0.64), Myc (4.28 fold, p = 0.0001), and RelA (1.48 fold, p = 0.019) were increased in UCP2 Tg mice after MCAO. There was no significant decrease in the anti-apoptotic gene Bcl2 (p = 0.226) after MCAO in UCP2 Tg mice. The cell cycle genes Ccng2 (−1.44 fold, p = 0.092) and Ccnh (−1.40 fold, p = 0.028) were all decreased after MCAO in UCP2 Tg mice. In summary, ischemia in wild-type mice increased the expression of inflammatory genes and suppressed the expression of anti-apoptotic and a few cell cycle genes. Overexpression of UCP2 ameliorated the increase in IL-6, the decrease in Bcl2, and increased the expression of cell cycle genes as well.

**Table 1:** Results of PCR Array.

Symbol	Full name	FoldDifference			FoldDifference	
		WT MCAO/WT sham	P value	TG MCAO/Sham	p value
**Bcl2**	**B-cell leukemia/lymphoma 2**	−1.98	0.021	−1.43	0.226
**Ccng2**	**Cyclin G2**	−1.17	0.031	−1.44	0.092
**Ccnh**	**Cyclin H**	−1.42	0.074	−1.40	0.028
**Chek1**	**Checkpoint kinase 1 homolog**	1.78	0.024	2.24	0.008
**Esr1**	**Estrogen receptor 1 alpha**	3.35	0.022	1.39	0.639
**Il6**	**Interleukin 6**	23.36	0.050	7.34	0.037
**Mcl1**	**Myeloid cell leukemia sequence 1**	−2.24	0.362	4.52	0.152
**Myc**	**Myelocytomatosis oncogene**	3.93	0.039	4.28	<0.001
**RelA**	**V-rel reticuloendothe-liosis viral oncogene homolog A**	1.77	0.046	1.48	0.019
**Tnf**	**Tumor necrosis factor**	20.19	<0.001	24.04	0.001

After being normalized against house-keeping gene, the average delta Ct values of targets genes were compared between the test samples and control samples (n = 4 in each group) and presented as fold change. Negative value indicates decrease and positive value indicates increase compared to control. Data were analyzed using RT^2^ Profiler™ PCR Array Data Analysis Excel Template provided by SuperArray.

### 2.4 Change in Cell Signaling Proteins

A protein array focused on p53 associated proteins was used to measure the change in protein levels in the ischemic penumbra between UCP2 Tg and wild-type mice after 1 hour MCAO and 24 hours of reperfusion. Protein levels were normalized to pan-cytokeratin before a comparison was made between the two groups (Table 2). In comparing UCP2 Tg to wild-type after MCAO, cell survival related proteins HSP-90, MEK, phospho-serine 473 AKT, protein p300/CBP, p300/CBP associated protein and protein kinase C were significantly increased. Cdc25A, which is a phosphotase mediating c-myc induced apoptosis, was decreased in UCP2 Tg mice. In addition, overexpression of UCP2 increased non-cleaved caspase 3 but decreased non-cleaved caspase 9 levels.

**Table 2:** P53 Protein Array.

Protein	UCP2 Tg	WT MCAO	p value
**Caspase-3**	8.56±1.95	5.82±0.64	0.001
Caspase-9**	5.82±0.39	6.75±0.46	0.038
**Cdc25a**	3.99±0.18	5.04±0.61	0.016
**Heat Shock Protein 90**	4.96±0.48	4.14±0.46	0.049
**JNK**	4.97±0.16	5.94±0.01	0.001
**MAP Kinase Kinase (MEK)**	8.24±1.13	5.73±0.47	0.006
**AKT Phospho Ser 473**	6.57±0.24	4.82±0.53	0.024
**p300/CBP associated factor (PCAF)**	7.13±0.34	5.97±0.41	0.035
**p300/CBP**	4.67±0.63	3.76±0.38	0.048
**Protein Kinase C**	8.92±1.36	6.36±0.77	0.016

Mean and s.d values are derived from 4 samples in each group after being normalized against the housekeeping protein. Data were analyzed using Data Analysis Workbook for the Panorama p53 Array provided by Sigma.

## Discussion

In this study, we first confirmed that overexpression of UCP2 is neuroprotective against transient focal ischemia induced brain damage, which is consistent to previous publication [12]. This protection was independent from any abnormalities in the cerebral vasculature. We then investigated the potential mechanisms of UCP2 mediated neuroprotection.

Decreases in inflammatory cytokines appear to contribute to the neuroprotection provided by UCP2. Transient focal ischemia caused 23 and 20 fold increases in IL-6 and Tnf-α mRNA respectively, suggesting that ischemia induces neuroinflammatory responses in the brain. Overexpression of UCP2 significantly reduced the IL-6 expression to less than one third of the levels observed in wild-type animals after being challenged with ischemia, suggesting that UCP2 suppresses neuroinflammation in the brain after stroke. Reports have shown that expression of IL-6 in human patients after ischemic stroke is associated with the size of infarction [27]. IL-6 is increased in brain injury and is directly up-regulated by IL-1β and TNF-α [28].Mice transgenically overexpressing the antioxidant enzyme glutathione peroxidase had reduced expression of IL-6 after transient focal ischemia [29]. It is possible that suppression of IL-6 lessens inflammatory response. This is supported by studies showing that UCP2/3 overexpressing mice have reduced inflammatory responses after endotoxin challenges and decreased IL-6 and IL-4 levels after lipopolysaccharide challenge [24] and that UCP2 homozygous knockout (UCP2^−/−^) mice have increased expression of IL-6 after listeria infection compared with wild-type controls [30].

Ischemia in wild-type mice suppressed Bcl2 and overexpression of UCP2 alleviated the suppression. Bcl2 is a well-known anti-apoptotic gene that inhibits the mitochondria-initiated cell death pathway. Bcl-2 provides protection from oxidative stress [31] and cerebral ischemia [32]. Transgenically overexpressing Bcl-2 reduced infarction from permanent focal ischemia [33]. Upregulation of Bcl-2 is associated with several neuroprotective strategies including ischemic pre-conditioning [34]. Our studies confirmed previous findings that cerebral ischemia reduces the transcript levels of Bcl-2 [35] and showed that upregulation of UCP2 in transgenic mice ameliorated ischemia-induced Bcl2 suppression. Similarly, Mcl-1, a Bcl-2 family protein [36], [37]. is suppressed by ischemia and its suppression is elevated by UCP2 overexpression. These data suggest that the neuroprotective effects of UCP2 may be associated with prevention of Bcl2 family suppression induced by cerebral ischemia. This is supported by the study showing that upregulation of UCP2 by ghrelin protects hypoxia-induced damage by increasing Bcl-2/Bax ratios [38].

Ischemia resulted in cell arrest as reflected by the suppression of cell cycle genes cyclin G2 and cyclin H,, which stimulate cell proliferation and survival [39], [40]. UCP2 overexpression alleviated ischemia-induced suppression on cyclin G2 and cyclin H, suggesting that the neuroprotective effects of UCP2 may be associated with cell cycle regulation.

Estrogen receptor alpha (Esr1) and checkpoint kinase 1 (Check1) are involved in neuroprotection, DNA repair and regulation of cell proliferation [40], [41]. In the present study, Esr1 increased after ischemia and reperfusion injury, suggesting ischemia activates cell protective machinery. However, since UCP2 did not increase Esr1, its neuroprotetive effects may not be associated with Esr1. Two oncogenes, Myc and RelA, are upregluated by ischemia. This is consistent with previous publication showing increased Myc in brain after cerebral ischemia [42]. While the role of these two genes in ischemic brain damage has not been established, persistent expression of Myc induces apoptosis [43]. RelA is a major component of NF-κB transcription factor [44]. Since Myc and RelA are both upregluated after focal ischemia in both wild-type and UCP2 Tg mice, UCP2 mediated neuroprotection may not be associated with these two genes.

UCP2 activates cell survival signaling pathways. The protein levels of HSP90, phospho-AKT, PKC, p300/CBP, p300/CBP associated factor (PCAF), and MEK were significantly increased in the ischemic penumbra of UCP2 Tg mice after transient focal ischemia compared to wild-type ischemic mice. Ischemic preconditioning is known to increase HSP90 [45]. Inhibiting HSP90 with geldanamycin increased oxidative stress and cell death in PC12 cells [46]. Neuroprotective hypothermia, ischemic pre-conditioning and ischemic post-conditioning cause phosphorylation of AKT 473 [17], [18] and PKC [47]. Inhibiting phosphorylation of AKT blocks the beneficial effects of hypothermia, ischemic pre- and post-conditioning [17], [18]. Upregulating UCP2 by ghrelin provides neuroprotection by activating ERK and PKC pathways [38]. MEK phosphorylates MAP kinase (ERK) and promotes cell proliferation. MEK protects from glutamate-induced damage in neuronal cultures [19]. Inhibiting MEK blocked neuroprotection by ischemic pre-conditioning [18]. p300/CBP plays a critical role in transcriptional regulation of hypoxia responsive genes, including hypoxia-inducing factor and its downstream vascular endothelial growth factor (VEGF) and erythropoietin [48]. Both VEGF and erythropoietin protect brain against ischemic injury [49], [50]. Our results suggest that UCP2 protects against ischemia-induced brain damage by activating cell survival signals.

UCP2 decreases protein levels of Cdc25A in the ischemic penumbra region. Cdc25A is a phosphatase with significant roles in cell cycle progression by activating cyclin dependent kinases [51]. It is regulated post-translational by the ubiquitin-proteasome pathway [52]. Cdc25A is necessary for c-myc induced apoptosis. Cdc25a activity is increased in degenerating neurons of patients who died with Alzheimer's disease [51]. Blocking Cdc25a inhibits c-myc mediated apoptosis [53]. UCP2 decreases the levels of Cdc25a, therefore it may reduce apoptotic cell death induced by ischemia.

The protein levels of caspase-3 increased and caspase-9 decreased in the ischemic penumbra of UCP2 Tg mice. Since the measured caspase-3 and caspase-9 are the uncleaved form, it is not clear whether the increase in caspase3 or decrease in capspase-9 has any pathogenic meaning.

Our results confirmed overexpressing UCP2 is neuroprotective from transient focal ischemia. Overexpressing UCP2 suppresses mRNA levels of inflammatory cytokine and elevated the ischemia-induced suppression of cell cycle genes. Furthermore, overexpression of UCP2 increases the protein levels of cell survival factors.
